# Chemotherapy-Induced Neutropenia and Febrile Neutropenia in the US: A Beast of Burden That Needs to Be Tamed?

**DOI:** 10.1093/oncolo/oyac074

**Published:** 2022-05-12

**Authors:** Ralph Boccia, John Glaspy, Jeffrey Crawford, Matti Aapro

**Affiliations:** Center for Cancer and Blood Disorders, Bethesda, MD, USA; UCLA School of Medicine, Los Angeles, CA, USA; Duke Cancer Institute, Duke University School of Medicine, Durham, NC, USA; Cancer Centre, Clinique de Genolier, Genolier, Switzerland

**Keywords:** febrile neutropenia, hospitalization, mortality, costs and cost analysis, US

## Abstract

Neutropenia and febrile neutropenia (FN) are common complications of myelosuppressive chemotherapy. This review provides an up-to-date assessment of the patient and cost burden of chemotherapy-induced neutropenia/FN in the US, and summarizes recommendations for FN prophylaxis, including the interim guidance that was recommended during the coronavirus disease 2019 (COVID-19) pandemic. This review indicates that neutropenia/FN place a significant burden on patients in terms of hospitalizations and mortality. Most patients with neutropenia/FN presenting to the emergency department will be hospitalized, with an average length of stay of 6, 8, and 10 days for elderly, pediatric, and adult patients, respectively. Reported in-hospital mortality rates for neutropenia/FN range from 0.4% to 3.0% for pediatric patients with cancer, 2.6% to 7.0% for adults with solid tumors, and 7.4% for adults with hematologic malignancies. Neutropenia/FN also place a significant cost burden on US healthcare systems, with average costs per neutropenia/FN hospitalization estimated to be up to $40 000 for adult patients and $65 000 for pediatric patients. Evidence-based guidelines recommend prophylactic granulocyte colony-stimulating factors (G-CSFs), which have been shown to reduce FN incidence while improving chemotherapy dose delivery. Availability of biosimilars may improve costs of care. Efforts to decrease hospitalizations by optimizing outpatient care could reduce the burden of neutropenia/FN; this was particularly pertinent during the COVID-19 pandemic since avoidance of hospitalization was needed to reduce exposure to the virus, and resulted in the adaptation of recommendations to prevent FN, which expanded the indications for G-CSF and/or lowered the threshold of use to >10% risk of FN.

## Teaser

Neutropenia and febrile neutropenia are common complications of myelosuppressive chemotherapy. This review assesses the effect on patients and the cost burden of chemotherapy-induced neutropenia and febrile neutropenia in the US, and summarizes recommendations for prophylaxis, including the interim guidance that was recommended during the COVID-19 pandemic.

**Implications for Practice**

In the US, chemotherapy-induced neutropenia/febrile neutropenia (FN) remain a significant burden on patients with cancer, in terms of hospitalization and mortality, and on healthcare systems, in terms of cost. This is particularly concerning during the ongoing coronavirus disease 2019 (COVID-19) pandemic where it is of utmost importance that neutropenia/FN are prevented to avoid additional hospital visits, which may increase exposure to COVID-19. Efforts to decrease the number and duration of hospital stays (eg, optimization of outpatient care, efficiency improvements in management processes, development of educational initiatives, and design of risk-stratification tools validated in clinical practice) could reduce the burden of neutropenia/FN.

## Introduction

Neutropenia and febrile neutropenia (FN) are common and potentially life-threatening complications of myelosuppressive chemotherapy.^1-3^ While there is no standard classification for neutropenia, a patient is generally considered neutropenic when their absolute neutrophil count (ANC) falls below 1.5 × 10^9^/L (1500/mm^3^).^4^ The severity of neutropenia can be categorized as mild (ANC, 1.0-1.5 × 10^9^/L), moderate (0.5-1.0 × 10^9^/L), severe (0.2-0.5 × 10^9^/L), or very severe (<0.2 × 10^9^/L, termed “agranulocytosis”).^4,5^ FN, considered an oncologic emergency, is defined by the Infectious Diseases Society of America, American Society of Clinical Oncology (ASCO), and National Comprehensive Cancer Network (NCCN) as an oral temperature ≥38.3°C (101.0°F), or a sustained temperature ≥38.0°C (100.4°F) for 1 hour, and an ANC <0.5 × 10^9^/L or an ANC that is expected to decrease to <0.5 × 10^9^/L within 48 hours.^6-8^ The European Society for Medical Oncology’s (ESMO) definition of FN states that a sustained temperature >38.0°C (100.4°F) must be present for 2 hours.^1^

In the US, incidence of drug-induced neutropenia is 2.4-15.4 cases/million each year^5,9^ and incidence of FN is 7.8 cases/1000 patients with cancer.^10^ In Europe, the rate of drug-induced neutropenia is 1.6-9.2 cases/million^5,11-15^ and the FN rate is ~8 cases/1000 patients.^1^ The ASCO 2015, ESMO 2016, and NCCN May 2021 guidelines on FN management outline factors associated with an increased FN risk, which include older age, advanced disease, poor performance status, a history of neutropenia/FN, infection, recent surgery or open wounds, prior chemotherapy, bone marrow involvement, and the presence of pulmonary, renal, hepatic, or cardiovascular comorbidities.^1,8,16^

Neutropenia/FN represent a significant burden to patients, predisposing them to serious and often life-threatening infections that can result in hospitalization and antibiotic treatment, reducing the intensity or delaying critical chemotherapy treatment (potentially shortening overall survival), and diminishing quality of life.^1,17-22^ A 2006 analysis noted that in-hospital mortality among 41 779 adults with cancer hospitalized for FN was 9.5%.^23^ Neutropenia/FN among patients with cancer also represent a significant burden on healthcare resources and costs, with direct costs driven by hospitalizations and treatment administration, and indirect costs driven by work loss, with differences in economic burden depending on cancer type.^24-26^

This review provides an up-to-date assessment of the burden of neutropenia/FN in patients with cancer, and the cost burden to US healthcare systems, although the findings are likely applicable to other developed regions. We also appraise recommendations from ASCO, ESMO, and NCCN for granulocyte colony-stimulating factor (G-CSF) prophylaxis of FN. Additionally, we evaluate the interim guidance from these societies on how FN management was adapted during the coronavirus disease 2019 (COVID-19) pandemic, an evolving international situation that placed new and unique pressures on the management of patients with cancer.

## Materials and Methods

### Aim

Using the available literature, a review was undertaken to evaluate and summarize reports of the burden of neutropenia/FN on patients with cancer (in terms of hospitalization and mortality) and US healthcare systems (in terms of cost) over the past 5 years. Following this, we reviewed recommendations from ASCO, ESMO, and NCCN for G-CSF prophylaxis of FN as well as the interim guidance from these societies on how FN management was adapted during the COVID-19 pandemic.

### Search Strategy

A PubMed search was conducted. The search strategy comprised the following string: (“neutropenia” OR “febrile neutropenia” OR “neutropenic fever”) AND (“United States” OR “America”) AND (“infection” OR “antibiotic” OR “hospital*” OR “dose reduction” OR “dose delay” OR “dose intensity” OR “relative dose intensity” OR “RDI” OR “quality of life” OR “QoL” OR “cost” OR “economic” OR “afford*” OR “resource” OR “burden”). All terms were mapped to medical subject heading terms. Eligible reports included US-based real-world studies (observational, retrospective, and prospective studies) and healthcare resource utilization/cost analyses. The search was restricted to full English-language articles published within the last 5 years (since January 1, 2015). In addition, a supplementary PubMed search was conducted to identify reports on the mortality of FN in patients with hematologic malignancies; only one eligible publication was identified.

Guidelines reviewed comprised the ASCO 2015 recommendations for the use of white blood cell growth factors, ESMO 2016 clinical practice guidelines on the management of FN, and NCCN guidelines on hematopoietic growth factors (version 4, 2021).^1,8,16^ Recommendations from these 3 societies in response to the COVID-19 pandemic were also reviewed.^27-29^

## Results

### Included Studies

Overall, 88 papers were identified. After title and abstract screen, 74 papers were excluded due to not being conducted in the US (*n* = 31), not reporting burden (hospitalization, mortality, or cost; *n* = 17), no mention of neutropenia/FN (*n* = 10), not in patients with cancer (*n* = 10), neutropenia/FN not caused by myelosuppressive chemotherapy (*n* = 5), and being a non-human study (*n* = 1). This left 14 eligible papers.

### Burden on Patients

#### Hospitalizations

Eleven studies published in the last 5 years reported findings on hospitalization rates and length of stay (LOS; Table 1). Overall, these papers analyzed over 670 000 and 118 000 hospitalizations or discharges for neutropenia/FN in children and adults, respectively.

**Table 1.** Burden to patients—hospitalization.

Design	Cohort	*N*	Hospitalization due to neutropenia/FN	Risk factors for hospitalization	LOS (days)	Risk factors for longer LOS	Ref
*All ages*							
Retrospective analysis of NEDS, 2006-2014	Patients with cancer (any age) presenting to ED with FN	348 868 ED visits for FN	94% of ED visits due to FN resulted in hospital admission	Older age Public versus private, self-pay, and other insurance Treatment at a metropolitan non-teaching or non-metropolitan hospital versus metropolitan teaching hospitals (*P* < .001)	—	—	Baugh et al^30^
*Pediatric pts*							
Retrospective analysis of NIS, 2007-2014	Pediatric pts with cancer hospitalized for FN	104 315 hospitalizations for FN	2007: 12.9 per 100 000 US population 2014: 18.1 per 100 000 US population	Age 5-9 (*P* < .001) Age 10-14 (*P* < .001) Male (*P* = .003) Midwest (*P* < .001) and Western regions (*P* < .001)	2007: 4.0^a^ 2014: 5.0^a^	Age 15-19 years versus 0-4 years (*P* = .02) Leukemia primary diagnosis (*P* < .001) Medicare/Medicaid insurance (*P* < .001) or other insurance (*P* = .003) versus private insurance Comorbid infections (sepsis, pneumonia, viral URI, meningitis, gastroenteritis, mycosis, and skin and subcutaneous infections; all *P* < .001)	Lekshminarayanan et al^31^
Retrospective analysis of NIS and KID, 2012	Pediatric (<18 years) and adult (≥18 years) pts with cancer hospitalized for neutropenia	16 859 pediatric cancer-related hospitalizations for neutropenia	Hospitalizations for neutropenia accounted for 22.7% of all cancer-related hospitalizations	Younger (0-9 vs 10-17 years; *P* < .001) Higher number of chronic conditions (*P* < .001) Admitted through the ED (*P* < .001)	8.5^b^	Leukemia primary diagnosis	Tai et al^32^
Retrospective analysis of Californian OSHPD database, 1983-2011	Pediatric pts (<18 years) with cancer hospitalized for FN	24 559 discharges	—	—	8.0^a^	Age <1, 1-4, 10-15, and 15-18 versus 5-9 years Treatment at PCSCs versus non-PCSCs (*P* < .0001) Black non-Hispanic, Hispanic, and Asian ethnicity versus Caucasian AML, bone tumors, or soft tissue sarcoma versus ALL Complications (sepsis/bacteremia, hypotension, pneumonia, fungal infections, other bacteremia infections)	Alvarez et al^33^
Cross-sectional analysis of KID, 2012	Pediatric pts with cancer admitted for FN	120 675 hospital discharges among pediatric pts with cancer	12.2% of discharges due to FN 16.3 per 100 000 US pediatric pts with cancer	Age 0-9 years Male Non-Hispanic white race Insured (private more common than public) ALL primary diagnosis	7.5^b^	Non-white race (Black or Hispanic) Other insurance versus private insurance	Mueller et al^34^
Cross-sectional analysis of KID, 2009	Pediatric pts with cancer admitted for FN	110 967 hospital discharges among pediatric pts with cancer	10.1% of discharges due to FN 13.4 per 100 000 US pediatric pts with cancer	Age 0-9 years Male Non-Hispanic white race Insured (private more common than public) ALL primary diagnosis	7.1^b^	—	Mueller et al^35^
Retrospective analysis of NEDS, 2006-2010	Pediatric pts with cancer with visits to the ED	294 289 weighted ED visits for pediatric pts with cancer	82.3% of ED visits due to FN resulted in hospital admission	—	—	—	Mueller et al^36^
*Adult and elderly pts*							
Retrospective cohort study of Medicare 20% sample data from the CMS, 2007-2015	Elderly pts (aged ≥66 years) with breast cancer, lung cancer, or NHL	2138 pts with breast cancer 3521 pts with lung cancer 2862 pts with NHL	Breast cancer: 88.1% (ICU: 20.4%) Lung cancer: 93.0% (ICU: 29.0%) NHL: 93.2% (ICU: 25.7%)	—	Breast cancer: 6.2 (ICU: 4.7)^b^ Lung cancer: 6.5 (ICU: 4.7)^b^ NHL: 6.8 (ICU: 5.5)^b^	—	Li et al^37^
Retrospective analysis of NIS and KID, 2012	Pediatric (<18 years) and adult (≥18 years) pts with cancer hospitalized for neutropenia	91 560 adult cancer-related hospitalizations for neutropenia	Hospitalizations for neutropenia accounted for 5.2% of all cancer-related hospitalizations	Younger (18-44 years; *P* < .001) Higher number of chronic conditions (*P* < .001) Admitted from the ED (*P* < .001) Private health insurance (*P* < .001) Treatment in an urban teaching hospital (*P* < .001)	9.6^b^	Leukemia primary diagnosis	Tai et al^32^
Retrospective analysis of a prospective study at the Washington University School of Medicine, 2013-2015	Adult pts with AML and MDS with infection-related SAEs after 10-day cycles of decitabine	85 adult pts with AML and MDS	65.7% (*n* = 163) of hospital admissions due to FN or infection	—	—	—	Ali et al^38^
Retrospective analysis of VACCR, 1998-2008	Adult pts with newly diagnosed DLBCL	522 pts with baseline body composition information	34.5% (*n* = 150) of hospital admissions due to FN	Baseline sarcopenia (*P* = .046) Higher comorbidity score (*P* < .001) Stage III/IV disease (*P* = .034) First-cycle ARDI ≥ 85% (*P* = .006)	—	—	Xiao et al^39^
Retrospective analysis of NIS, 2009-2011	Adult pts with breast cancer hospitalized for FN	26 628 FN hospitalizations	—	—	5.7^b^	—	Pathak et al^20^

Median.

Mean.

Abbreviations: ALL, acute lymphoblastic leukemia; AML, acute myeloid leukemia; ARDI, average relative dose intensity; CMS, Centers for Medicare & Medicaid Services; DLBCL, diffuse large B-cell lymphoma; ED, emergency department; FN, febrile neutropenia; ICU, intensive care unit; KID, Kids’ Inpatient Database; LOS, length of stay; MDS, myelodysplastic syndrome; NEDS, Nationwide Emergency Department Sample; NHL, non-Hodgkin lymphoma; NIS, National Inpatient Sample; OSHPD, Office of Statewide Health Planning and Development; PCSC, Pediatric Cancer Specialty Center; pts, patients; URI, upper respiratory infection; VACCR, Veteran’s Health Administration Central Cancer Registry.

For patients with cancer who visit the emergency department (ED) for FN, nearly all visits end in hospitalization. Among pediatric patients, it has been reported that 82.3% of visits resulted in hospitalization,^36^ increasing to 94.0% in one study of patients of any age.^30^

Among pediatric patients with cancer, 10.1%-22.7% of hospitalizations were due to neutropenia/FN.^32,34,35^ Patient-related factors found to increase the likelihood of hospitalization included younger age (especially <10 years old), male, non-Hispanic white ethnicity, and having private rather than public health insurance.^31,32,34,35^ Disease-related risk factors consisted of primary diagnosis of acute lymphoblastic leukemia and higher numbers of chronic conditions.^32,34,35^ Moreover, a higher risk of hospitalization was observed in patients admitted through the ED, and those presenting to institutions in the Midwest or Western regions.^31,32^ The finding that younger children are at a higher risk of hospitalization reflects that hematologic malignancies, especially leukemias, are typically diagnosed in young children and in these patients bone marrow suppression is caused not only by chemotherapy but also by the cancer itself originating in the bone marrow.^32,35^ Indeed, hospitalization for FN was found to be common among pediatric patients with acute lymphoblastic leukemia.^34,35^ The observation that hospital admission for neutropenia/FN was more common through the ED also aligns with the urgent need to immediately treat patients with broad-spectrum antibiotics to try and combat the causative infectious agent.^32^ Moreover, the finding that hospitalization for FN is more likely to occur in institutions based in Midwestern or Western regions could be due to a number of factors including differences in the management protocols within institutions and/or healthcare systems, as well as factors such as patient travel distance to hospital and ease of access.^34,35^

Among adults, one retrospective analysis of over 90 000 hospitalizations estimated that neutropenia accounted for 5.2% of all cancer-related hospitalizations.^32^ In studies of adult patients with (AML)/myelodysplastic syndromes (MDS) or diffuse large B-cell lymphoma (DLBCL), 65.7% and 34.5% of hospital admissions were due to FN, respectively.^38,39^ A number of patient- and disease-related factors reported to increase the likelihood of hospitalization for neutropenia/FN were in-line with those listed in FN management guidelines^1,8,16,40^ such as older age, presence of comorbidities, advanced stage disease, and first cycle chemotherapy average relative dose intensity ≥85%.^30,32,39^ Although, one study reported that younger age (18-44 years) was associated with an increased risk of hospitalization.^32^ This finding may have been influenced by other characteristics of the patient population (eg, cancer type and number of chronic conditions). Baseline sarcopenia was an additional factor found to increase the risk of hospitalization for FN.^39^ However, the older age of sarcopenic versus non-sarcopenic patients (mean, 68.1 vs 61.2 years) may have contributed to this finding.^39^ Findings on type of insurance were conflicting with one study reporting that private insurance was associated with hospitalization,^32^ whereas another found that hospitalization was more likely among those with public insurance.^30^ Increased likelihood of hospitalization with public insurance is expected due to less resources available for outpatient management. Similar to pediatric patients, admittance from the ED was again associated with increased risk of hospitalization for FN in adult patients.^32^ One study reported a higher likelihood of hospitalization in urban teaching hospitals^32^ whereas another found that hospitalization was more common in metropolitan non-teaching or non-metropolitan hospitals.^30^ Again, these differences are potentially due to variations in the management protocols used and patient-related factors.

In elderly patients (aged ≥66 years), inpatient hospital care was needed for 2121 of 2407 (88.1%) cases of FN in patients with breast cancer, 3571 of 3840 (93.0%) in patients with lung cancer, and 3342 of 3587 (93.2%) in patients with non-Hodgkin lymphoma (NHL).^37^ A larger proportion of patients with NHL (28.3%) and lung cancer (16.7%) was aged ≥80 years than those with breast cancer (9.9%), and patients with breast cancer had fewer comorbid conditions than those with lung cancer or NHL, possibly contributing to the higher rates of inpatient hospital care.^37^

The average LOS of patients admitted to hospital for FN was 4.0-8.5, 5.7-9.6, and 6.2-6.8 days for children, adults, and elderly patients, respectively.^20,31-35,37^ The shorter LOS among pediatric and elderly patients is likely due to these patients often being admitted on a precautionary basis and once observed briefly are then dismissed. Although for elderly patients this could also be due to higher mortality rates.

Longer LOS in children was associated with both younger (<1-4 years) and older (10-19 years) age, non-Caucasian race, and having public insurance rather than private insurance.^31,33,34^ The finding regarding public insurance was speculated to be due to pressure on hospitals to discharge patients quickly when the patients’ insurance is private due to having to justify the stay.^33^ Pediatric patients with leukemia, bone tumors, or soft tissue sarcomas, as well as comorbid infections or hypotension had longer LOS.^31-33^ Longer LOS was also common among adult patients with leukemia.^32^ Regarding institution-related factors, one Californian retrospective analysis found that median LOS was significantly longer at pediatric cancer specialty centers compared with non-specialty centers (9 vs 7 days; *P* < .0001).^33^ The authors hypothesized that this may reflect the increased severity of illness seen at specialist centers as well as the increased likelihood that patients at specialist centers may initiate their next cycle of chemotherapy while still an inpatient once their neutrophil counts have returned to normal.^33^

#### Mortality

Eight studies, 5 in children with cancer, 2 in adults with solid tumors, and 1 in adults with hematologic malignancies, reported in-hospital mortality rates (Table 2). Among pediatric patients with cancer hospitalized for neutropenia/FN, mortality rates ranged from 0.4% to 0.8%,^31,32,34,35^ with one study reporting a higher rate of 3%.^33^ A significantly higher risk of mortality was observed among adolescents aged 15-19 years versus children aged 0-4 years, and in patients with infections (eg, mycosis, meningitis, pneumonia, and sepsis).^31^ The authors hypothesized that the disease biology may be different in adolescent compared with younger patients, contributing to unfavorable outcomes.^31^ Other factors which potentially could have contributed to greater mortality included variation in care, social support, delays in seeking care, and socioeconomic status.^31^

**Table 2.** Burden to patients—mortality.

Design	Cohort	*N*	Mortality rate	Risk factors for mortality	Ref
*Pediatric pts*					
Retrospective analysis of NIS, 2007-2014	Pediatric pts with cancer hospitalized for FN	104 315 hospitalizations for FN	0.8%	Age 15-19 years versus 0-4 years (*P* = .002) Comorbid infection (mycosis *P* < .001; meningitis *P* = .01; pneumonia *P* < .001; sepsis *P* < .001)	Lekshminarayanan et al^31^
Retrospective analysis of NIS and KID, 2012	Pediatric (<18 years) and adult (≥18 years) pts with cancer hospitalized for neutropenia	16 859 pediatric cancer-related hospitalizations for neutropenia	0.6%	—	Tai et al^32^
Retrospective analysis of Californian OSHPD database, 1983-2011	Pediatric pts (<18 years) with cancer hospitalized for FN	24 559 discharges	3.0%	—	Alvarez et al^33^
Cross-sectional analysis of KID, 2012	Pediatric pts with cancer admitted for FN	120 675 hospital discharges among pediatric pts with cancer	0.5%	—	Mueller et al^34^
Cross-sectional analysis of KID, 2009	Pediatric pts with cancer admitted for FN	110 967 hospital discharges among pediatric pts with cancer	0.4%	—	Mueller et al^35^
*Adult and elderly pts*					
Retrospective case-control study conducted at the UMHS, 2006-2016	Elderly pts (≥60 years) diagnosed with AML	48 elderly pts with AML treated with FLAG 45 elderly pts with AML treated with CLO	—	Longer duration of neutropenia potentially contributed to a 7-times higher 30-day induction mortality with CLO versus FLAG	Scappaticci et al^41^
Retrospective analysis of UHC database, 2004-2012	Adult pts (≥18 years old) with solid tumors hospitalized for FN	61 086 adult pts with solid tumors hospitalized for FN	7.0%	Lung cancer versus other solid tumors (*P* < .0001) Sepsis Pneumonia Infection ICU admission Presence of comorbidities	Cupp et al^42^
Retrospective chart review, 2010-2014	Adult inpatients with hematologic malignancies and FN	244 FN events in 216 pts	7.4%	Sepsis Elevated bilirubin	Butts et al^43^
Retrospective analysis of NIS, 2009-2011	Adult pts with breast cancer hospitalized for FN	26 628 FN hospitalizations	2.6%	Age ≥65 years versus <65 years (*P* < .05)	Pathak et al^20^

Abbreviations: AML, acute myeloid leukemia; CLO, clofarabine-based induction; FLAG, fludarabine, cytarabine, and granulocyte colony-stimulating factor; FN, febrile neutropenia; ICU, intensive care unit; KID, Kids’ Inpatient Database; NIS, National Inpatient Sample; OSHPD, Office of Statewide Health Planning and Development; pts, patients; UHC, University Health Consortium; UMHS, University of Michigan Health System.

Among adults with cancer hospitalized for neutropenia/FN, mortality rate ranged between 2.6% and 7.0% for those with solid tumors^20,42^ and was 7.4% in a retrospective chart review of patients with hematologic malignancies.^43^ Risk factors for mortality among patients with solid tumors included older age (≥65 vs <65 years), lung cancer versus other solid tumors, the presence of comorbidities, infection, sepsis, or pneumonia, and admission to intensive care.^20,42^ The higher mortality for patients with lung cancer is likely due to these patients being older than patients with other solid tumors, with 50.0% and 31.6% of patients aged ≥65 years, respectively.^42^ In addition, patients with lung cancer were more likely to have 2 or more comorbidities, and comorbid heart and lung diseases, than patients with other solid tumors.^42^ The presence of sepsis or elevated bilirubin were risk factors for mortality among patients with hematologic malignancies.^43^ An additional retrospective case-control study in elderly patients (aged ≥60 years) with AML reported a significantly longer duration of grade III/IV neutropenia with clofarabine-based induction, which was hypothesized to contribute to a 7-times higher 30-day induction mortality rate compared with fludarabine, cytarabine, and G-CSF (FLAG).^41^

#### Burden on healthcare systems

Table 3 summarizes the findings on the cost of neutropenia/FN care in patients with cancer; 4 of the 6 studies were for treatment of pediatric patients. In an analysis of 2012 data from the National Inpatient Sample, total costs of hospitalizations for neutropenia among adult patients with cancer amounted to $2.3 billion.^32^ In pediatric patients, 2 cross-sectional analyses of the KID demonstrated that the financial burden of FN care rose from $587 million in 2009 to $881 million in 2012.^34,35^ However, another study using 2012 KID data estimated total costs to only be $439 million.^32^ This difference in costs despite using the same database is likely due to methodological differences such as the age of included patients (≤17 vs ≤19 years), how cancer-related neutropenia hospitalizations were identified (ICD-9-CM based on previous literature vs clinical classification software codes), and how costs were reported (2012 USD vs conversion from 2012 USD to 2015 USD dollar using the consumer price index).^32,34^ The mean cost per FN hospitalization was $20 000-40 000 for adults^20,32^ and $8000-65 000 for children^31,32,34,35^ with cancer.

**Table 3.** Burden to healthcare systems.

Design	Cohort	*N*	Cost per FN episode	Total cost	Risk factors for higher cost	Ref
*Pediatric pts*						
Retrospective analysis of NIS, 2007-2014	Pediatric pts with cancer hospitalized for FN	104 315 hospitalizations for FN	2007: $8771^a^ 2014: $11 202^a^	—	Age 10-14 and 15-19 years versus 0-4 years (*P* < .001) Leukemia primary diagnosis (*P* < .001) Medicare/Medicaid insurance (*P* = .001) or other insurance (*P* < .001) versus private insurance Comorbid infections (sepsis, pneumonia, viral URI, meningitis, gastroenteritis, mycosis, and skin and subcutaneous infections; all *P* < .001) Western region (*P* = .03)	Lekshminarayanan et al^31^
Retrospective analysis of NIS and KID, 2012	Pediatric (<18 years) and adult (≥18 years) pts with cancer hospitalized for neutropenia	16 859 pediatric hospitalizations for neutropenia	$20 366^b^	$439 million (27.2% of all cancer-related hospitalization costs)	Leukemia primary diagnosis	Tai et al^32^
Cross-sectional analysis of KID, 2012	Pediatric pts with cancer admitted for FN	120 675 hospital discharges among pediatric pts care with cancer	$65 536^b^	~$881 million	—	Mueller et al^34^
Cross-sectional analysis of KID, 2009	Pediatric pts with cancer admitted for FN	110 967 hospital discharges among pediatric pts with cancer	$52 160^b^	~$587 million	—	Mueller et al^35^
*Adult and elderly pts*						
Retrospective cohort study of Medicare 20% sample data from the CMS, 2007-2015	Elderly pts (aged ≥66 years) with breast cancer, lung cancer, or NHL	2138 pts with breast cancer 3521 pts with lung cancer 2862 pts with NHL	Breast cancer: $13 457^b^ Lung cancer: $15 409^b^ NHL: $16 009^b^	—	—	Li et al^37^
Retrospective analysis of NIS and KID, 2012	Pediatric (<18 years) and adult (≥18 years) pts with cancer hospitalized for neutropenia	91 560 adult hospitalizations for neutropenia	$20 778^b^	$2.3 billion (8.3% of all cancer-related hospitalization costs)	Leukemia primary diagnosis	Tai et al^32^
Retrospective analysis of NIS, 2009-2011	Adult pts with breast cancer hospitalized for FN	26 628 FN hospitalizations	$37 087^b^	—	—	Pathak et al^20^

Median.

Mean.

CMS, Centers for Medicare & Medicaid Services; FN, febrile neutropenia; KID, Kids’ Inpatient Database; NHL, non-Hodgkin lymphoma; NIS, National Inpatient Sample; pts, patients; URI, upper respiratory infection.

Factors significantly associated with higher costs for pediatric patients included older age (10-19 years), non-private insurance payers, and comorbid infection.^31^ In one study, a primary diagnosis of leukemia in both adults and children was associated with a higher mean cost per neutropenia hospitalization compared with other cancer types.^32^ Within this study, patients with leukemia had the longest mean LOS compared with other cancer types, potentially contributing to the higher costs of neutropenia care.^32^ Among elderly patients, mean total costs of care per FN episode were reported to be highest in patients with NHL (~$15 000) and lowest for those with breast cancer (~$12 000).^37^ The higher cost of FN care for patients with NHL was again potentially due to these patients having the longest mean LOS compared with patients with breast or lung cancer.^37^ Accounting for all but 1% of mean total costs, inpatient care was the largest driver of cost for FN care.^37^ Costs associated with outpatient-only care for FN were far lower than costs for inpatient care ($849 for breast cancer, $841 for lung cancer, and $1322 for NHL) but care in this setting occurred much less frequently.^37^

#### FN prophylaxis with G-CSF in patients with cancer: current guidelines

Evidence-based guidelines from societies based in the US (ASCO and NCCN) and Europe (ESMO) align in their recommendations and advise that G-CSFs, such as filgrastim and pegfilgrastim, should be used as prophylaxis for FN in patients with cancer who are at high risk of FN and receiving myelosuppressive chemotherapy.^1,8,16^ G-CSF is recommended as primary prophylaxis when a chemotherapy regimen carries a high risk (≥20%) of FN and in patients with an intermediate risk (10-20%) if they have ≥1 risk factor.^1,8,16^

Presently, the guidelines generally do not cover targeted agents. These newer agents have more precise mechanisms of action than chemotherapy but many are still myelosuppressive, including monoclonal antibodies (eg, rituximab), immunomodulatory drugs (eg, lenalidomide), and kinase inhibitors (eg, cyclin-dependent kinase 4/6 inhibitors), many of which are used in metastatic or relapsed/refractory disease.^44^ In the May 2021 release of the NCCN guidelines on hematopoietic growth factors, for a number of regimens recommendations for G-CSF support apply with or without the addition of monoclonal antibodies (eg, trastuzumab, rituximab).^8^ There is the potential for increased neutropenia risk with the addition of monoclonal antibodies; for example, rituximab has been associated with prolonged neutropenia with or without chemotherapy.^8,45^

#### FN prophylaxis with G-CSF in patients with cancer: COVID-19 considerations

In response to the COVID-19 pandemic, ASCO, ESMO, and NCCN released guidance on G-CSF administration to reduce the risk of drug-induced neutropenia/FN and associated infection in patients with cancer, and ensure that their treatment can be delivered at the most effective dose and on time (Table 4).^27-29^ All 3 interim guidelines recommended that the indications for G-CSF were expanded and/or the threshold of G-CSF use was lowered to >10% risk, to reduce the risk of FN and the numbers of patients requiring treatment in hospitals or emergency settings, thereby decreasing COVID-19 exposure.^27-29^ These statements were concordant with pre-COVID-19 recommendations advocating the use of G-CSF prophylaxis in special circumstances for patients at intermediate risk of FN (10%-20%).^1,8^

**Table 4.** Interim recommendations for management of FN in patients with cancer receiving chemotherapy during the coronavirus (COVID-19) pandemic.

Society	Recommendations
ASCO^27^	• G-CSF should be used cautiously and in line with guidelines from ASCO • To avoid neutropenia or myelosuppression, which may put the patient at higher risk of COVID-19 infection, prophylactic G-CSF would still be justified • Limited/no data available in patients with active COVID-19 needing G-CSF for neutropenia/FN • Decisions need to be based on the clinical situation • Regarding COVID-19, ASCO identifies 2 key areas of management of patients with potential FN: ◦ Prophylaxis—potential use of G-CSF in patients at a lower level of expected risk (eg, >10% risk) in order to reduce the risk of FN and emergency care; neutrophil count monitoring and regular contact advised ◦ Acute care—for patients with potential FN, evaluation of status should occur by phone/telemedicine to determine if the patient should be assessed in the clinic or sent to the ED. For those with known FN, standard guidelines^46^ for care (including isolation) should be followed irrespective of COVID-19 status. If available, rapid COVID-19 testing should be used to ascertain the level of PPE required for caregivers as well as the appropriate facility location for continued care. If rapid testing is unavailable, the patient should be managed for FN per standard guidelines with the assumption of COVID-19 infection
NCCN^29^	• Recommendations aim to minimize risk of hematologic complications associated with chemotherapy, reducing the need for hospital occupancy or additional infusion clinic/ED visits • Recommended that routine prophylactic G-CSF should be made available to all patients receiving intermediate- or high-risk chemotherapy regimens • Prophylactic G-CSFs may also be appropriate in patients receiving low-risk regimens when age or comorbidities increase their risk of FN • For patients experiencing FN who have not received prior prophylactic therapy with PEGylated G-CSFs, it is advised they start G-CSFs to shorten time to neutrophil recovery • For patients with respiratory infection, respiratory symptoms, or a confirmed/suspected COVID-19 infection and FN, G-CSF is not recommended due to the potential for increasing pulmonary inflammation and inflammatory cytokine (eg, IL-6) production associated with severe COVID-19 infection • Self-administration of daily filgrastim or long-acting pegfilgrastim (1-3 days after chemotherapy) or use of an on-body injector pegfilgrastim are recommended to minimize visits to outpatient centers and reduce the risk of COVID-19 exposure
ESMO^28^	• For patients with solid tumors not treated for cure, consider administering regimens at low risk of FN ◦ For use of regimens with a higher risk of FN, there must be considerable evidence that clearly outweighs potential emergency intervention and COVID-19 exposure • G-CSF indication should be expanded to include patients receiving chemotherapy with a lower risk of FN (the theoretical concern of acute respiratory failure due to G-CSF-induced leukocyte recovery in patients with COVID-19 pulmonary infection does not outweigh the benefit); however, this approach may require additional outpatient clinic visits • For outpatient management of FN in patients with lower risk, well-documented and verified criteria are available (eg, the MASCC FN risk group stratifications^47^), with published randomized trials using oral antibiotics • Use of antibiotic prophylaxis and/or prescription of stand-by antibiotics should be expanded due to a potential risk of a delay to emergency visits for patients who develop fever • Critical review and reduction of the use of steroids is recommended, if possible • In patients receiving a fluoropyrimidine, genetic testing to identify patients with DPD deficiency is recommended^48^ • No evidence is currently available demonstrating that neutropenia due to PARP or CDK4/6 inhibitors results in an increase in associated viral infections

ASCO, American Society of Clinical Oncology; CDK, cyclin-dependent kinase; COVID-19, coronavirus disease 2019; DPD, dihydropyrimidine dehydrogenase; ED, emergency department; ESMO, European Society for Medical Oncology; FN, febrile neutropenia; G-CSF, granulocyte colony-stimulating factor; IL-6, interleukin 6; MASCC, Multinational Association of Supportive Care in Cancer; NCCN, National Comprehensive Cancer Network; PARP, poly(adenosine diphosphate-ribose) polymerase; PEG, polyethylene glycol; PPE, personal protective equipment.

The NCCN recommended that self-administration of filgrastim or long-acting pegfilgrastim, or on-body injector pegfilgrastim should be considered to reduce the risk of contracting COVID-19 during outpatient visits.^29^ In patients with potential FN, ASCO recommended that the neutropenic status of patients should be first evaluated by telemedicine or phone to help decide if the patient should be assessed in the clinic or sent to the ED.^27^ In patients with confirmed FN, rapid COVID-19 testing should be used, if available, to inform the appropriate location for continued care and the level of personal protective equipment needed for caregivers.^27^ ESMO noted that the risk of acute respiratory failure due to G-CSF-induced leukocyte recovery in patients with COVID-19-associated lung infections did not outweigh the benefits of using G-CSF.^28^ Expanded use of antibiotics was recommended, alongside G-CSF, because of the risk of a delay to emergency visits during the pandemic for patients with fever.^28^ Regimens that are unlikely to induce FN were advised for patients with solid tumors undergoing non-curative treatment.^28^

## Discussion

This review of recent real-world evidence from the US indicates that chemotherapy-induced neutropenia/FN place a significant burden on patients with cancer, in terms of hospitalizations and mortality, and on healthcare systems, in terms of cost. Most patients presenting to the ED with neutropenia/FN will be hospitalized,^30,36,37^ with the average LOS being 6 days for elderly patients, ~8 days for children, and up to 10 days for adults.^20,32-34,37^ In this review, mortality rates for children with cancer hospitalized for neutropenia/FN were 0.4%-3.0%,^32-35^ with rates of 2.6%-7.0% reported in adults with solid tumors^20,42^ and 7.4% in adults with hematological malignancies.^43^ Improved awareness of factors that increase mortality during FN-related hospitalization (eg, older age and infection^20,31,42^) may help healthcare professionals improve survival by allowing earlier identification of those at risk and provide more individualized care.^42^

As well as directly affecting patient outcomes, neutropenia/FN place a significant cost burden on healthcare systems in the US and worldwide. In 2012, total hospitalization costs for neutropenia/FN in the US amounted to >$2 billion for adults and up to $880 million for children with cancer.^32,34^ Mean cost per hospitalization was reported to be up to $15 000 for elderly patients (2007-2015 data),^37^ $40 000 for adults (2009-2011 data),^20^ and $65 000 for children (2012 data).^34^

While restricting our search to articles published within the past 5 years to give contemporary estimates of FN, a limitation of this review is that several papers focus on population databases with data gathered between 2005 and 2015. Differences in methods between studies, such as patient classification ages for pediatric and elderly patients, makes comparing data and drawing conclusions more complex. Furthermore, due to a lack of a single discharge code for FN, several of the included studies identified cases of neutropenia/FN in patients with cancer using combinations of diagnostic codes, which may be subject to error, identify patients with neutropenia caused by means other than chemotherapy, and select a sample biased toward sicker patients who are more likely to be hospitalized. Some of the databases used in the included studies do not track revisits and therefore some patients may have been counted more than once. Although providing insight into direct costs of care for neutropenia/FN, the included studies did not assess societal costs including productivity loss and patient and caregiver time and transportation costs. However, strengths of the included studies include their representation of real-world clinical practice and inclusion of large numbers of patients, providing an updated overview of the burden of neutropenia/FN on healthcare systems and patients with cancer.

Efforts are needed to decrease hospital stays, thereby reducing the burden of neutropenia/FN on patients and healthcare systems. Outpatient FN treatment was found to be far less costly than inpatient care, but also far less common.^37^ Outpatient management of FN can be optimized through the knowledge and experience of physicians and pharmacists, who can identify suitable patients for oral antibiotic therapy and monitor treatment.^46,49^ The development of educational initiatives for patients with cancer, their caregivers, and healthcare professionals can also decrease the burden of neutropenia/FN by informing them on how to reduce the risk of neutropenia-related infections and enabling them to proactively treat infection.^50^ This can be provided by physicians, pharmacists, oncology nurses, and physician assistants, who can also contribute to the development of and adherence to their institution’s guidelines and processes.^49,51^ FN management can also be improved through the design and implementation of more efficient processes, to overcome barriers preventing the timely administration of antibiotics.^1,52,53^

The Multinational Association for Supportive Care in Cancer (MASCC) risk index score was developed to identify patients with FN at low risk of serious complications or death, and guidelines recommend that it can be used to identify patients that can be managed in the outpatient setting.^1,7,47^ However, application of MASCC score does not appear to be resulting in fewer hospitalizations. Real-world experience indicates that the MASCC score is too slow to be used in real-life emergency medicine and is inaccurate, with a predictive value of only 83%.^30,54^ The Clinical Index of Stable Febrile Neutropenia (CISNE) is a newer score found to be more accurate than MASCC and more appropriate for use in the emergency setting.^55-57^ However, CISNE only applies to solid tumors and is not recommended for use in patients presenting with septic shock or a previously known severe infection. Continued research is required to develop and validate risk stratification tools that can quickly and accurately identify patients at highest risk of complications from FN in real time in clinical practice, while being mindful of cost-effectiveness and patient quality of life.

Evidence-based guidelines recommend that G-CSFs are used as prophylaxis for FN in patients with cancer receiving a high-risk (≥20%) chemotherapy regimen, or an intermediate-risk (10%-20%) regimen if ≥1 risk factor is present.^1,8,16^ In response to the COVID-19 pandemic, ASCO, ESMO, and NCCN released guidance on G-CSF administration to reduce the infection risk in patients with cancer at risk of FN. The recommendations suggested that the indications for G-CSF were expanded and/or the threshold of G-CSF use was lowered to >10% risk of FN.^27-29^ Guidelines recommend that filgrastim is administered subcutaneously at a dose of 5 μg/kg/day starting 1-3 days after chemotherapy until recovery of neutrophils to normal levels.^1,8,16^ Pegfilgrastim should be given subcutaneously as a single 6 mg dose the day after myelosuppressive chemotherapy.^1,8,16^ When taken according to guideline recommendations, filgrastim and pegfilgrastim are equally effective.^58^ However, in clinical practice filgrastim is often under-dosed and therefore pegfilgrastim demonstrated more effectiveness than filgrastim in terms of reducing the incidence of FN and hospitalization, and achieving target dose intensity of chemotherapy.^58^ The most common adverse event with G-CSF therapy is bone pain, and patients should be encouraged to report this, together with any other adverse effects, to their treatment team.^1,8,16^ Usually, bone pain is managed with standard analgesics including acetaminophen and nonsteroidal anti-inflammatory drugs.^1,8,16^ Despite the use of prophylactic G-CSF and other management strategies, the impact of chemotherapy-induced myelosuppression on patients remains significant.^59^ Improving communication between healthcare professionals and their patients is crucial to bettering patients’ understanding of chemotherapy-induced myelosuppression and encouraging shared decision-making in regards to treatment.^59^ Biosimilars provide lower-cost alternatives to their reference medicines.^60,61^ Use of G-CSF biosimilars may improve costs of care and help reduce the burden of neutropenia/FN to healthcare systems.^62^

## Conclusion

In the US and indeed globally, chemotherapy-induced neutropenia/FN remain a significant burden on patients with cancer in terms of hospitalization and mortality, and on healthcare systems in terms of cost. This burden may be reduced through efforts to decrease the number and duration of hospital stays via the optimization of outpatient care, efficiency improvements in management processes, development of educational initiatives, and design of risk-stratification tools validated in clinical practice.

## Data Availability

The data underlying this article will be shared on reasonable request to the corresponding author.
